# Effect of Substitutional Pb Doping on Bipolar and Lattice Thermal Conductivity in p-Type Bi_0.48_Sb_1.52_Te_3_

**DOI:** 10.3390/ma10070763

**Published:** 2017-07-06

**Authors:** Hyun-sik Kim, Kyu Hyoung Lee, Joonyeon Yoo, Jehun Youn, Jong Wook Roh, Sang-il Kim, Sung Wng Kim

**Affiliations:** 1 Materials R&D Center, Samsung Advanced Institute of Technology, Samsung Electronics, Suwon 16419, Korea; projet.special@gmail.com (H.-s.K.); roh5397@gmail.com (J.W.R.); 2 Department of Nano Applied Engineering, Kangwon National University, Chuncheon 24341, Korea; khlee2014@kangwon.ac.kr; 3 Department of Materials Science and Engineering, University of Seoul, Seoul 02504, Korea; srw321@uos.ac.kr (J.Y.); schne92@uos.ac.kr (J.Y.); 4 Department of Energy Science, Sungkunkwan University, Suwon 16419, Korea

**Keywords:** thermoelectrics, bipolar conduction, lattice thermal conductivity, bismuth telluride

## Abstract

Cation substitutional doping is an effective approach to modifying the electronic and thermal transports in Bi_2_Te_3_-based thermoelectric alloys. Here we present a comprehensive analysis of the electrical and thermal conductivities of polycrystalline Pb-doped p-type bulk Bi_0.48_Sb_1.52_Te_3_. Pb doping significantly increased the electrical conductivity up to ~2700 S/cm at *x* = 0.02 in Bi_0.48-*x*_Pb*_x_*Sb_1.52_Te_3_ due to the increase in hole carrier concentration. Even though the total thermal conductivity increased as Pb was added, due to the increased hole carrier concentration, the thermal conductivity was reduced by 14–22% if the contribution of the increased hole carrier concentration was excluded. To further understand the origin of reduction in the thermal conductivity, we first estimated the contribution of bipolar conduction to thermal conductivity from a two-parabolic band model, which is an extension of the single parabolic band model. Thereafter, the contribution of additional point defect scattering caused by Pb substitution (Pb in the cation site) was analyzed using the Debye–Callaway model. We found that Pb doping significantly suppressed both the bipolar thermal conduction and lattice thermal conductivity simultaneously, while the bipolar contribution to the total thermal conductivity reduction increased at high temperatures. At Pb doping of *x* = 0.02, the bipolar thermal conductivity decreased by ~30% from 0.47 W/mK to 0.33 W/mK at 480 K, which accounts for 70% of the total reduction.

## 1. Introduction

The thermoelectric (TE) effect offers the direct conversion of a temperature gradient into electrical energy, and vice-versa. Among the many TE materials, (Bi,Sb)_2_(Te,Se)_3_-based alloys (p-type (Bi,Sb)_2_Te_3_ and n-type Bi_2_(Te,Se)_3_) have been intensively investigated because they exhibit the highest TE performance near room temperature [1,2]. However, current TE applications are limited due to low conversion efficiency, which is evaluated from a dimensionless TE figure of merit, *zT* = *S*^2^·*σ*·*T*/*κ* (*S*: Seebeck coefficient, *σ*: electrical conductivity, and *κ*: thermal conductivity) at a given absolute temperature (*T*). The *zT* value can be improved by reducing the *κ* and enhancing the *S* while maintaining the *σ*. Low *κ* maintains the temperature difference between the hot and cold sides of a material, and high *S* associates the high voltages generated at a certain temperature gradient.

*zT* can be enhanced by reducing the lattice thermal conductivity (*κ_latt_*) caused by interface phonon scattering in nanostructured materials, which effectively scatter low-frequency phonons. For example, Poudel et al. [1] reported a peak *zT* of 1.4 at 100 °C in nanograin composite Bi_0.5_Sb_1.5_Te_3_. Additionally, it has been recently found that dense dislocations formed at grain boundaries can intensify phonon scattering by the additional scattering of mid-frequency phonons [3]. Substitutional doping is another effective approach to reducing the *κ_latt_* by introducing point defects for phonon scattering, which target high-frequency phonons. It has been experimentally found that certain elements, such as Al, Ga, In, Cu, Ag, and Fe, reduce *κ_latt_* effectively [4,5,6,7,8]. However, the substitutional doping approach is inherently accompanied with the modification of electronic structure, resulting in the variation of power factor (*σ*·*S*^2^) and bipolar conduction.

We have recently demonstrated that the substitutional doping of Pb in Bi_0.48_Sb_1.52_Te_3_ can reduce *κ_latt_* and increase power factor (*σ*·*S*^2^) [9]. In this study, the TE transport properties, including *S*, *σ*, and *κ_latt_* of Pb-doped Bi_0.48_Sb_1.52_Te_3_ polycrystalline samples, were further analyzed by using the single parabolic band (SPB) model [10] and Callaway model [11], and compared with a Bi_0.48_Sb_1.52_Te_3_ polycrystalline sample in order to closely examine the effect of Pb substitution on bipolar conduction and *κ_latt_* reduction. The *S* and *σ* were fitted to experimental values by adjusting the deformation potentials and the effective masses of both the conduction and valence bands by the two-parabolic band model based on the SPB model. Through this model, the bipolar conduction (*κ_bp_*) was estimated, while the *κ_latt_* was analyzed using the Callaway model.

## 2. Results and Discussion

**Figure 1:**
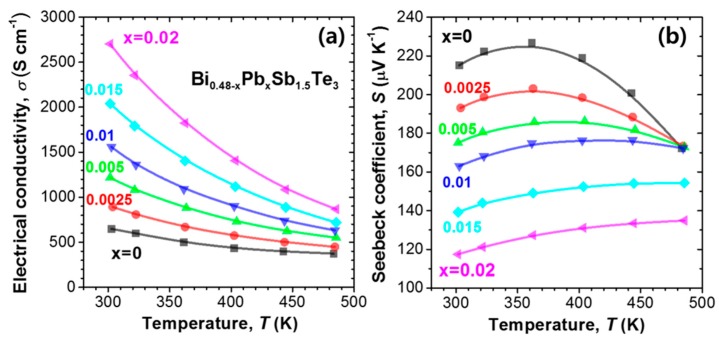
Temperature dependence of (**a**) electrical conductivity (*σ*) and (**b**) Seebeck coefficient (*S*) for Bi_0.48-*x*_Pb*_x_*Sb_1.52_Te_3_ (*x* = 0, 0.0025, 0.005, 0.01, 0.015, and 0.02). The *σ* and *S* data were taken from Reference [9].

The temperature dependence of *σ* and *S* of the Pb-doped samples (Bi_0.48-*x*_Pb*_x_*Sb_1.52_Te_3_, *x* = 0.0025, 0.005, 0.01, 0.015, and 0.02) and undoped Bi_0.48_Sb_1.52_Te_3_ (*x* = 0) is shown in Figure 1. The value of *σ* of Bi_0.48_Sb_1.52_Te_3_ at 300 K is about 640 S/cm, which increased significantly to 2700 S/cm (*x* = 0.02) with an increase in Pb content. The hole concentrations (*N_p_*) at 300 K were 2.47, 3.54, 4.96, 6.60, 8.76, and 11.96 × 10^19^/cm^3^ for *x* = 0, 0.0025, 0.005, 0.01, 0.015, and 0.02, respectively. Therefore, the substitution of Pb for Sb induces significant hole carriers by the introduction of acceptor defects. The value of *S* decreased from 215 μV/K to 117 μV/K, revealing a clear trade-off relationship with the *σ* value. It is noteworthy that while the maximum value of *S* is obtained at 360 K for *x* = 0, it shifts to a higher temperature of 480 K for *x* = 0.02; this implies that the charge compensation from bipolar conduction is reduced due to the significant increase in *N_p_* as Pb is added. 

**Figure 2:**
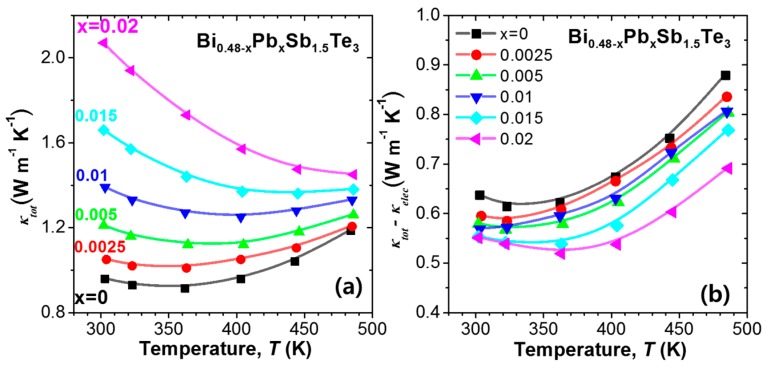
Temperature dependence of (**a**) total thermal conductivity (*κ_tot_*) and (**b**) (*κ_tot_*-*κ_elec_*) for Bi_0.48-*x*_Pb*_x_*Sb_1.52_Te_3_ (*x* = 0, 0.0025, 0.005, 0.01, 0.015, and 0.02). The *κ_tot_* data are taken from [9].

Figure 2 shows the temperature dependence of *κ_tot_* and (*κ_tot_*-*κ_elec_*) of the Pb-doped samples (Bi_0.48-_*_x_*Pb*_x_*Sb_1.52_Te_3_, *x* = 0.0025, 0.005, 0.01, 0.015, and 0.02) and undoped Bi_0.48_Sb_1.52_Te_3_ (Bi_0.48-*x*_Pb*_x_*Sb_1.52_Te_3_, *x* = 0), where *κ_tot_* is the measured total thermal conductivity and *κ_elec_* is the electronic thermal conductivity without considering the bipolar conduction. Thus, (*κ_tot_*-*κ_elec_*) is the thermal conductivity excluding the contribution of the increased *N_p_*. The *κ_elec_* was estimated using the Wiedemann–Franz law, and the Lorenz number was calculated using Equation (1) [12] which is deduced under the assumption of a single parabolic band (plus acoustic phonon scattering),

(1) $$L=1.5+\exp\left(-\frac{S}{116}\right).$$

Indeed, the *κ_tot_* increased with Pb content due to a significantly enhanced *N_p_* value (Figure 2a). As seen in Figure 2b, the (*κ_tot_*-*κ_elec_*) value decreased by over 14% to 0.55 W/mK for *x* = 0.02, as compared to 0.64 W/mK of the undoped sample at 300 K. Furthermore, the amount of reduction in the (*κ_tot_*-*κ_elec_*) value increased with increasing temperature and reduced up to 22% for *x* = 0.02 at 480 K. For *x* = 0.02, which is only a 1% substitution for the cation site, a significant reduction of 14–22% in (*κ_tot_*-*κ_elec_*) was achieved.

**Figure 3:**
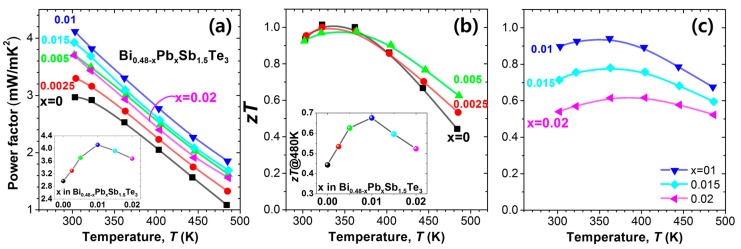
(**a**) Temperature dependence of power factor (*σS*^2^) for Bi_0.48-*x*_Pb*_x_*Sb_1.52_Te_3_ (*x* = 0, 0.0025, 0.005, 0.01, 0.015, and 0.02). Inset shows the power factor value at 300 K for the samples; (**b**,**c**) Temperature dependence of a dimensionless figure of merit, *zT*, for Bi_0.48-*x*_Pb*_x_*Sb_1.52_Te_3_ for (**b**) *x* = 0, 0.0025, and 0.005 and (**c**) *x* = 0.01, 0.015, and 0.02. Inset in (**b**) shows the *zT* value at 480 K. The power factor and *zT* data are taken from [9].

Figure 3a shows the temperature dependence of power factor, which shows ~40% increase to 4.11 mW/mK^2^ for *x* = 0.01 from 2.97 mW/mK^2^ for *x* = 0. The *N_p_* value of 4.96 × 10^19^/cm^3^ at *x* = 0.01 seems to be the optimum value for the highest power factor. For *x* = 0.015 and 0.02, the power factor decreased due to a significant reduction in the *S* value. Figure 3b,c exhibit *zT* values of the samples. The *zT* values at temperatures above 400 K increased as the substitution increased to *x* = 0.01, as shown in the inset of Figure 3b. For *x* = 0.02, the *zT* value reduced up to 440 K due to a significant increase in the *σ* value, which results in a significant rise in *κ_tot_*. The optimal *σ* range is 700–1200 S/cm for a high *zT* in this case.

Pb substitutional doping significantly increases *σ* due to an increase in *N_p_* and reduces the (*κ_tot_*-*κ_elec_*) value, which includes the bipolar thermal conductivity (*κ_bp_*) and lattice thermal conductivity (*κ_latt_*). In order to further understand the origin of (*κ_tot_*-*κ_elec_*) reduction, we first estimated the *κ_bp_* from a two parabolic band model, which is an extension of the SPB model that considers acoustic phonon scattering. Thereafter, the contribution of additional point defect scattering to *κ_latt_* was closely analyzed using the Debye–Callaway model and compared with the point defect contribution from its native cation disorder. The Pb substitutes are additional point defects in the native cation disorder of (Bi_0.48_Sb_1.52_) in their mother compound Bi_0.48_Sb_1.52_Te_3_.

In the two-band model, the thermoelectric parameters of valence and conduction bands computed from the Boltzmann transport equations [10] can be substituted into Equations (2)–(5),

(2) $$\sigma_{total}=\sum_{i}\sigma_{i},$$

(3) $$S_{total}=\frac{\sum_{i}S_{i}\sigma_{i}}{\sum_{i}\sigma_{i}},$$

(4) $$\;R_{H}{}_{total}=\frac{\sum_{i}R_{H}{}_{i}\sigma_{i}{}^{2}}{{\left(\sum_{i}\sigma_{i}\right)}^{2}},$$

(5) $$\kappa_{tot}=\;\kappa_{elec}+\;\kappa_{latt}+\;\kappa_{bp}=L\sigma T+\kappa_{latt}+\left(\sum_{i}S_{i}{}^{2}\sigma_{i}-S_{total}^{2}\sigma_{total}\right)T.$$

Here, *σ_i_*, *S_i_*, and *R_Hi_* in Equations (2)–(5) are the electrical conductivity, Seebeck coefficient, and Hall coefficient of an individual band, respectively. The parameters with subscript “*total*” include contributions from all the participating bands (these variables can be measured directly). Equation (5) describes the contribution of *κ_elec_*, *κ_latt_*, and *κ_bp_* to the *κ_tot_*. In order to estimate *κ_bp_*, the calculated *σ_total_* and *S_total_* of one sample were fitted to the experimental *σ* and *S* of the sample by adjusting the deformation potentials and density-of-states (DOS) effective masses (m*) of its valence and conduction bands (Table 1). Since it is nontrivial to extract a single band’s contribution to TE transport (band parameters) experimentally, we estimated each band’s contribution via modelling. For this purpose, we referred to the values of m* and the mobility of Bi_2_Te_3_ reported in literature. For example, the m* of holes (or electrons) in Bi_2_Te_3_ reported in the literature can elucidate the band structure of Bi_0.48_Sb_1.52_Te_3_ when two bands (valence and conduction) are assumed to participate in the transport. Due to the high crystal symmetry of Bi*_x_*Sb_2-*x*_Te_3_, more than one pocket of Fermi surface contributes to m* as m* = *N_V_*^2/3^m_b_*, where *N_V_* and m_b_* are the valley (pocket) degeneracy and band mass of a single valley, respectively. The *N_V_* of the highest valence band of Bi*_x_*Sb_2-*x*_Te_3_ is 6, while that of the lowest conduction band is 2, as listed in Table 1 [13]. Similarly, the mobility of holes and electrons in Bi_2_Te_3_ reported in the literature along with m* can help us estimate reasonable deformation potentials. Moreover, *R_Hi_* was calculated from the measured Hall carrier concentration (=1/(*e R_Htotal_*)) and other band parameters computed above (deformation potential and m*). The Fermi level of each band obtained from *R_Hi_* was used to crosscheck that it calculated from *S_i_*. Because the band gap between the valence and conduction bands are given, we only needed to calculate the Fermi level of either band.

**Figure 4:**
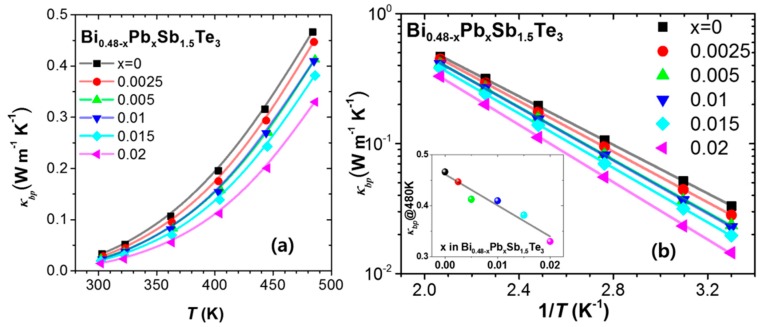
(**a**) Temperature dependence of bipolar thermal conductivity, *κ_bp_*, of Bi_0.48-*x*_Pb*_x_*Sb_1.52_Te_3_ (*x* = 0, 0.0025, 0.005, 0.01, 0.015, and 0.02) estimated from the two-parabolic band model [10]; (**b**) ln(*κ_bp_*) vs. (1/*T*). Inset shows that the *κ_bp_* at 480 K decreases as the Pb substitution increases.

The estimated *κ_bp_* is plotted in Figure 4a,b. In Figure 4b, the plot of ln(*κ_bp_*) vs. (1/*T*) reveals that the bipolar conduction increases exponentially with temperature. It is clearly seen that the estimated *κ_bp_* decreases systematically with Pb doping, as inferred from Figure 1a and Figure 2. The inset in Figure 4b shows that the *κ_bp_* decreases by ~30% from 0.47 W/mK to 0.33 W/mK at 480 K for *x* = 0.02. The Pb doping of Bi_0.48_Sb_1.52_Te_3_ effectively suppresses the *κ_bp_* by increasing the concentration of the major carrier (holes) and reducing the concentration of the minor carrier (electrons), which partly accounts for the reduced *κ_latt_* values at high temperatures in Pb-doped Bi_0.48_Sb_1.52_Te_3_.

The estimated *κ_bp_* for each sample was added to the theoretical *κ_latt_*, which is calculated below, to compare with the experimentally determined *κ_latt_*. Heat flow (*q*) is defined as a product of thermal conductivity (*κ*) and temperature gradient (Δ*T*). The negative sign of the product (*q* = −*κ ΔT*) indicates that heat flows from hot to cold. *κ* can be expressed as arising from the heat capacity (*C_V_*), velocity (*v*), and distance between the collisions (*l*) according to the kinetic gas model presented in Equation (6).

(6) $$\kappa =\;\frac{1}{3}C_{V}vl=\frac{1}{3}C_{V}v^{2}\tau .$$

The *l* in Equation (6) is a product of *v* and the relaxation time, *τ*. Here, *κ* can also be described in terms of *C_V_*, *v*, and *τ* (Equation (6)). By applying Equation (6) to the phonons in solids instead of gas particles, we obtain the Callaway equation for *κ_latt_* (Equation (7)).

(7) $$\kappa_{latt}=\;\frac{1}{3}\int_{0}^{\omega_{max}}C_{V}\left(\omega \right)v_{g}{\left(\omega \right)}^{2}\tau \left(\omega \right)d\omega .$$

Because each parameter is dependent on frequency (*ω*), the product of each parameter is integrated over the frequency. Here, *v_g_* refers to the phonon group velocity (*v_g_* = *dω*/*dk*). By treating the *v_g_* and phase velocity (*v_p_* = *ω*/*k*) as equivalent to the speed of sound, *v*, Equation (7) can be written as Equation (8) (Debye–Callaway model).

(8) $$\kappa_{latt}=\;\frac{k_{B}}{2\pi^{2}v}{\left(\frac{k_{B}T}{\hbar }\right)}^{3}\int_{0}^{\theta_{a}/T}\frac{\tau_{\mathrm{total}}\left(z\right)\;z^{4}e^{z}}{{\left(e^{z}-1\right)}^{2}}dz,$$

where *k*_B_, *ћ*, *θ*, and *z* are the Boltzmann constant, reduced Planck’s constant, Debye temperature *θ_a_*, and *ћω*/*k_B_T*, respectively [11]. The values of Debye temperature *θ_a_* (94 K) [14] and average phonon velocity (2147 m/s) [15] were obtained from experimental literature data. The *κ_latt_* of a material can be calculated using Equation (8) once its *τ*_total_ (*z*) is determined from the individual relaxation times (*τ_i_*) for different scattering processes, according to Matthiessen’s rule (Equation (9)),

(9) $$\tau_{\mathrm{total}}{\left(z\right)}^{-1}=\;\sum_{i}\tau_{i}{\left(z\right)}^{-1}=\;\tau_{U}{\left(z\right)}^{-1}+\;\tau_{B}{\left(z\right)}^{-1}+\;\tau_{PD}{\left(z\right)}^{-1}.$$

The parameter for relaxation times is associated with Umklapp scattering (*τ_U_*), boundary scattering (*τ_B_*), and point-defect scattering (*τ_PD_*). Here, point-defect scattering arises from atomic disorders in alloys, and is described in terms of a scattering parameter (*Г*) within the *τ_PD_* formula as:

(10) $$\tau_{PD}{}^{-1}=P\;f\left(1-f\right)\;\omega^{4}=\;\frac{V\omega^{4}}{4\pi v^{3}}Г,$$

and

(11) $$\text{Г}=f\left(1-f\right)\left[{\left(\frac{∆M}{M}\right)}^{2}+\frac{2}{9}{\left\{\left(G+6.4\gamma \right)\frac{1+r}{1-r}\right\}}^{2}{\left(\frac{∆a}{a}\right)}^{2}\right].$$

In Equations (10) and (11), P is the fitting parameter and *Г* is a scattering parameter related to the difference in mass (Δ*M*) and lattice constant (Δ*a*) between two constituents of an alloy. The parameters *f*, Δ*M*, *G*, γ*, r*, and Δ*a* are the fractional concentration of either constituents, difference in mass, parameter representing a ratio of the fractional change of bulk modulus to that of local bond length, Grüneisen parameter, Poisson ratio, and the difference in lattice constant, respectively. The parameter (*G*) represents material dependent (Δ*K/K*) (*R/*Δ*R*), where Δ*K* and Δ*R* are the changes in bulk modulus and local bond length, respectively. We used the same parameters used in reference [16] for estimating the relaxation times for Umklapp scattering (*τ_U_*) and boundary scattering (*τ_B_*).

**Figure 5:**
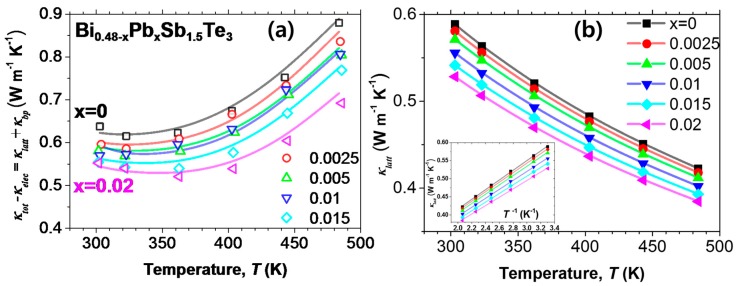
(**a**) (*κ_tot_*-*κ_elec_*) values of the samples (Figure 2b) with fitted values (lines); (**b**) Calculated value (*κ_latt_* estimated by the Callaway model). The further 10% reduction is achieved by Pb doping (*x* = 0.02). Inset shows the linear relationship between *κ_latt_* and (1/T).

For estimating the relaxation time for point-defect scattering (*τ_PD_*), we regard *P* and *f* (or P f(1 −f)) in Equation (10) as adjustable parameters in the calculation; these parameters were fitted to experimental *κ_latt_* varying with *f* for the (Bi_1-*f*_Sb*_f_*)_2_Te_3_ alloy by Stordeur and Sobotta [17] to model the *κ_latt_* of the undoped sample in Figure 2b with *f* = 0.48/2 = 0.24 (Bi fraction in the cation site). The P and P f(1−f) were fitted to 28.19 × 10^−41^ s^3^ and 5.142 × 10^−41^ s^3^ and the result is represented as a black solid line in Figure 5. For the Pb-doped samples, the additional scattering due to Pb point defects was estimated with separated relaxation time for Pb disorder, *τ_PD_*_(Pb)_, using the fractional concentration of Pb in the cation site, which is *f_Pb_* = *x*/2 (0.0013, 0.0025, 0.005, 0.0075, and 0.001 for *x* = 0.0025, 0.005, 0.01, 0.015, and 0.02, respectively). The fitting parameter for the scattering of Pb defects, P_Pb_, is 208.8 × 10^−41^ s^3^, and thus, the $P_{\mathrm{Pb}}f_{\mathrm{Pb}}\left(1-f_{\mathrm{Pb}}\right)$ values are 0.229, 0.521, 1.039, 1.553 and 2.067 × 10^−41^ s^3^ for *x* = 0.0025, 0.005, 0.01, 0.015, and 0.02, respectively (Table 2). The solid lines in Figure 5 exhibit the results of the calculation to which the estimated *κ_bp_* was added. It is noteworthy that *P*_Pb_ is ~7.4 times higher than *P* for its native cation disorder. The effect of Pb substitution on *κ_latt_* reduction appears to be much greater than that of the native cation disorder between Bi and Sb. It seems that the interaction of disorders within the matrix, for example, bonding nature and structure, is important to the inducing of strong point-defect scattering, thus influencing the local vibration mode around the point defects.

Although no meaningful enhancement in the maximum *zT* was observed since Pb doping induced excessive hole carriers, it was evident that Pb doping effectively suppressed both *κ_latt_* and *κ_bp_* simultaneously. The bipolar contribution to the thermal conductivity decreased by ~30% from 0.47 W/mK to 0.33 W/mK at 480 K for *x* = 0.02, while the bipolar contribution to the reduction in total thermal conductivity increased at higher temperatures. At 480 K, the contribution of bipolar conduction suppression to thermal conductivity reduction increased up to 0.13 W/mK, which is 70% of the total reduction of 0.19 W/mK, due to Pb doping (*x* = 0.02). Therefore, the substitutional doping approach should be considered not only for the introduction of additional point defects for phonon scattering, but also for the suppression of bipolar conduction in Bi*_x_*Sb_1-*x*_Te alloys, especially at high temperatures.

## 3. Materials and Methods

High-purity elemental Bi (99.999%, 5N Plus), Sb (99.999%, 5N Plus), Te (99.999%, 5N Plus), and Pb (99.999%, Sigma Aldrich, St. Louis, MO, USA) were used as starting materials. According to the formula, the mixed elements were loaded into a quartz tube and vacuum-sealed under 10^−4^ Torr. The stoichiometrically mixed elements were vacuum sealed and the contents were melted in a box furnace for 10 h at 1273 K, and then water quenched. The ingots were ground in a ball mill and consolidated by spark plasma sintering (SPS) using a graphite die (diameter = 10.4 mm) under a dynamic vacuum with the application of 50 MPa uniaxial pressure at 753 K. Room temperature Hall measurements were carried out in a constant magnetic field (1 T) and electric current (50 mA) using a Keithley 7065 system. The *S* and *σ* values were measured using a ZEM-3 system (M8, Advanced RIKO, Inc., Yokohama, Japan) in the temperature range from 300 K to 480 K. The *κ* values (*κ* = *ρ*_s_*C*_p_*λ*) were calculated from separated measurements of sample density (*ρ*_s_), heat capacity (*C*_p_), and thermal diffusivity (*λ*). The *C*_p_ values obtained using a Quantum Design physical property measurement system were almost constant at ~0.187 J·g^−1^·K^−1^, and the *λ* values were collected by a laser-flash method (TC-9000, Advanced RIKO, Inc., Yokohama, Japan). The TE properties were measured perpendicular to the SPS press direction for polycrystalline Bi_0.48_Sb_1.52_Te_3_ and Pb-doped Bi_0.48_Sb_1.52_Te_3_ samples.

## 4. Conclusions

In this study, the electrical and thermal conductivities and Seebeck coefficient of a series of polycrystalline bulk Pb-doped p-type Bi_0.48_Sb_1.52_Te_3_ were comprehensively analyzed using the single parabolic band model and Debye–Callaway model, and compared with that of Bi_0.48_Sb_1.52_Te_3_ polycrystalline alloys to closely examine the effect of Pb substitution on bipolar conduction and lattice thermal conductivity. A two-band model based on the single parabolic band model showed that Pb doping effectively suppressed the bipolar thermal conduction. The bipolar contribution to thermal conductivity decreased by ~30% from 0.47 W/mK to 0.33 W/mK at 480 K for *x* = 0.02 in Bi_0.48-*x*_Pb*_x_*Sb_1.52_Te_3_. By using the Debye–Callaway model, it was found that the phonon scattering effect of Pb substitutes was much greater than the native alloy disorder in Bi_0.48_Sb_1.52_Te_3_. The interaction of disorders with the matrix, for example, bonding nature and structure, appeared to be important in inducing strong point-defect scattering. Therefore, we conclude that Pb doping is effective in suppressing both the bipolar thermal conduction and lattice thermal conductivity simultaneously in Bi_0.48_Sb_1.52_Te_3_ alloys. In addition, the bipolar contribution to thermal conductivity reduction increased at high temperatures in Pb doping. At 480 K, the contribution of bipolar conduction suppression to thermal conductivity reduction increased up to 0.13 W/mK, which is 70% of the total reduction of 0.19 W/mK, due to Pb doping (*x* = 0.02).

## Figures and Tables

**Table 1:** Band parameters used to estimate the bipolar contribution to thermal conductivity, *κ_bp_*, of the Pb-doped samples using the two-band model.

Band Parameters	Bi_0.48_Sb_1.52_Te_3_	Bi_0.48-*x*_Pb*_x_*Sb_1.52_Te_3_				
		*x* = 0.0025	*x* = 0.005	*x* = 0.01	*x* = 0.015	*x* = 0.002
Valence band (VB) *E_def_* ^†^ (eV)	14.9	14.4	13.6	13.4	12.3	12.2
VB m* (in *m*_0_) ^‡^	1.30	1.35	1.41	1.46	1.47	1.48
VB N*_V_* ^§^	6	6	6	6	6	6
Conduction band (CB) *E_def_* ^†^ (eV)	13.8	12.5	11.5	10.0	9.1	8.2
CB m* (in *m*_0_) ^‡^	0.84	0.84	0.84	0.84	0.84	0.84
CB *N_V_* ^§^	2	2	2	2	2	2
No. of acceptors (10^−19^ cm^−3^)	2.47	3.53	4.96	6.60	8.76	11.96
Band gap (eV)	0.145	0.145	0.145	0.145	0.145	0.145
*C_l_* (GPa) ^‖^	54.7	54.7	54.7	54.7	54.7	54.7

^†^ *E_def_* = deformation potential. ^‡^ m* = density-of-states effective mass (*m*_0_ = electron mass). ^‖^ *C_l_* = longitudinal elastic constant. ^§^ *N_V_* = number of valley degeneracy.

**Table 2:** Point defect contributions to the total relaxation rate ($\tau_{\mathrm{total}}{}^{-1}$) used to model *κ_latt_* of the samples.

Sample	$\tau_{total}{}^{-1}$	*Fitting Parameter* P (10^−41^ s^3^)	*Fitting Parameter* $P_{Pb}\;$(10^−41^ s^3^)	Pf(1−f)(10^−41^ s^3^), *f* = 0.24	$P_{Pb}f_{Pb}\left(1-f_{Pb}\right)$ (10^−41^ s^3^)
Bi_0.48_Sb_1.52_Te_3_	$\tau_{U}{}^{-1}+\tau_{B}{}^{-1}+\tau_{PD}{}^{-1}$	28.19	-	5.142	-
Bi_0.48-*x*_Pb*_x_*Sb_1.52_Te_3_	$\tau_{U}{}^{-1}+\tau_{B}{}^{-1}+\tau_{PD}{}^{-1}+\tau_{PD}{}_{\left(\mathrm{Pb}\right)}^{-1}$	28.19	208.8	5.142	0.229
*x* = 0.0025					
*f*_Pb_ = 0.0011					
Bi_0.48-*x*_Pb*_x_*Sb_1.52_Te_3_	$\tau_{U}{}^{-1}+\tau_{B}{}^{-1}+\tau_{PD}{}^{-1}+\tau_{PD}{}_{\left(\mathrm{Pb}\right)}^{-1}$	28.19	208.8	5.142	0.521
*x* = 0.005					
*f*_Pb_ = 0.0025					
Bi_0.48-*x*_Pb*_x_*Sb_1.52_Te_3_	$\tau_{U}{}^{-1}+\tau_{B}{}^{-1}+\tau_{PD}{}^{-1}+\tau_{PD}{}_{\left(\mathrm{Pb}\right)}^{-1}$	28.19	208.8	5.142	1.039
*x* = 0.01					
*f*_Pb_ = 0.005					
Bi_0.48-*x*_Pb*_x_*Sb_1.52_Te_3_	$\tau_{U}{}^{-1}+\tau_{B}{}^{-1}+\tau_{PD}{}^{-1}+\tau_{PD}{}_{\left(\mathrm{Pb}\right)}^{-1}$	28.19	208.8	5.142	1.553
*x* = 0.015					
*f*_Pb_ = 0.0075					
Bi_0.48-*x*_Pb*_x_*Sb_1.52_Te_3_	$\tau_{U}{}^{-1}+\tau_{B}{}^{-1}+\tau_{PD}{}^{-1}+\tau_{PD}{}_{\left(\mathrm{Pb}\right)}^{-1}$	28.19	208.8	5.142	2.067
*x* = 0.02					
*f*_Pb_ = 0.01					
